# Three-dimensional holographic visualization of high-resolution myocardial scar on HoloLens

**DOI:** 10.1371/journal.pone.0205188

**Published:** 2018-10-08

**Authors:** Jihye Jang, Cory M. Tschabrunn, Michael Barkagan, Elad Anter, Bjoern Menze, Reza Nezafat

**Affiliations:** 1 Department of Medicine (Cardiovascular Division), Beth Israel Deaconess Medical Center and Harvard Medical School, Boston, MA, United States of America; 2 Department of Computer Science, Technical University of Munich, Munich, Germany; 3 Division of Cardiovascular Medicine, University of Pennsylvania, Philadelphia, PA, United States of America; Universitatsklinikum Wurzburg, GERMANY

## Abstract

Visualization of the complex 3D architecture of myocardial scar could improve guidance of radio-frequency ablation in the treatment of ventricular tachycardia (VT). In this study, we sought to develop a framework for 3D holographic visualization of myocardial scar, imaged using late gadolinium enhancement (LGE), on the augmented reality HoloLens. 3D holographic LGE model was built using the high-resolution 3D LGE image. Smooth endo/epicardial surface meshes were generated using Poisson surface reconstruction. For voxel-wise 3D scar model, every scarred voxel was rendered into a cube which carries the actual resolution of the LGE sequence. For surface scar model, scar information was projected on the endocardial surface mesh. Rendered layers were blended with different transparency and color, and visualized on HoloLens. A pilot animal study was performed where 3D holographic visualization of the scar was performed in 5 swines who underwent controlled infarction and electroanatomic mapping to identify VT substrate. 3D holographic visualization enabled assessment of the complex 3D scar architecture with touchless interaction in a sterile environment. Endoscopic view allowed visualization of scar from the ventricular chambers. Upon completion of the animal study, operator and mapping specialist independently completed the perceived usefulness questionnaire in the six-item usefulness scale. Operator and mapping specialist found it useful (usefulness rating: operator, 5.8; mapping specialist, 5.5; 1–7 scale) to have scar information during the intervention. HoloLens 3D LGE provides a true 3D perception of the complex scar architecture with immersive experience to visualize scar in an interactive and interpretable 3D approach, which may facilitate MR-guided VT ablation.

## Introduction

Late gadolinium enhancement (LGE) imaging in cardiovascular magnetic resonance (CMR) is a clinical gold standard for imaging of the myocardial scar [1]. LGE has been used to identify extent, volume, and characteristics of myocardial scar in patients with prior myocardial infarction [1, 2]. In particular, the detailed scar depiction of the LGE has been suggested to be useful for planning and guiding scar-related ventricular tachycardia (VT) ablation by characterizing location and extent of the scar [3, 4].

In the assessment of the VT substrate, understanding the 3D scar architecture is particularly important. Voltage mapping is performed to identify “conduction channels” in and around the scar that could represent the VT isthmus. In the histological study, conduction channels have been described as surviving fiber bundles embedded in myocardial scar areas that can be located at any level of the myocardial wall, with a variable thickness and 3D structure [5, 6]. 3D LGE provides a better understanding of the complex scar anatomy compared with voltage mapping alone for guiding the radio-frequency (RF) ablation for VT [3]. Therefore, visualization of the 3D scar is important for identification of VT substrate and guidance of RF ablation for VT treatment.

Augmented reality has been emerging for visualizing complex medical data, particularly during and planning medical procedures [7]. In particular, HoloLens (Microsoft, Redmond, WA) [8] has enabled visualization of complex medical data that can provide immersive experience to interactively explore the data in 3D space in the mixed-reality environment, and has been tested in various clinical scenarios [9, 10]. Augmented reality allows true 3D visualization that provides 3D depth perception, and opens up the possibility for interacting with the medical data without physical contact to assist while maintaining sterile environment and reduce the risk of infection.

In this study, we sought to develop a framework for 3D holographic visualization of the LGE images on augmented reality HoloLens to provide a true 3D perception of the scar architecture with a direct physician interaction in a sterile environment. A pilot animal study was performed to demonstrate the feasibility of using 3D HoloLens visualization of scar in electrophysiology study.

### Related works

#### LGE for guidance of VT ablation

In structural heart disease, VT is usually caused by re-entry, involving an arrhythmogenic substrate in the region of ventricular scar from a prior myocardial infarction [11]. The extent of LGE has been reported to identify the arrhythmogenic substrate [12], and improves a risk stratification for implantable cardioverter defibrillator therapy [13, 14] in patients with ventricular arrhythmias. Scar border zone defined in LGE has shown good correlation with VT inducibility [15] and spontaneous ventricular arrhythmias [16–18]. Therefore, various studies used LGE for planning and guiding VT ablation by integrating LGE into clinical electroanatomic mapping system [3, 4]. Recent studies showed improved outcome of using MRI-guided VT ablation compared to the mapping only system [19, 20].

#### AR for guidance of interventional procedures

Augmented reality (AR) shows promise for supporting interventional procedures. After the first in-human example of creation of dynamic interactive holograms from intraprocedural data [21], various studies have explored the use of holographic augmentation in various interventional procedures such as invasive structural cardiac procedures [22], ultrasound-guided transcatheter aortic valve implantation [23], neurosurgery [24], endovascular intervention [9], and near-infrared fluorescence based image guided surgery [10]. In the field of electrophysiology, a recent study reports first in-human experience of projecting intra-procedural electrophysiology data into an interactive holographic display [25].

#### Stereoscopic 3D visualization of the anatomy

Stereoscopic visualization improves our understanding of the complex 3D structures. In the conventional 2D flat screen visualization, 3D objects are projected onto the 2D flat screen, and users must visualize the complex 3D structures in their minds to fully understand the geometry and their relations [26, 27]. 3D stereoscopic presentation of images allow having additional depth information and make it easier to detect diagnostically relevant shapes, orientations, and relative positions of anatomical features [28]. A recent study compared virtual 3D stereoscopic model vs. 2D flat screen projected images when learning the head and neck vascular anatomy, and showed that 3D stereoscopic model offers more effective and increased ability to correctly identify the anatomy [29].

#### Touchless interaction in operating room

Touchless human-computer interaction to augment operator’s experience in sterile environment is important in interventional and surgical procedures [30]. Various techniques have been developed for touchless interaction in the operating room by using stereo camera [31, 32], voice commands [33, 34], and structured-light sensor [35]. HoloLens allows a combination of gesture recognition and voice command which offers physicians high degree of flexibility in interacting during the intervention.

## Materials and methods

**Fig 1:**
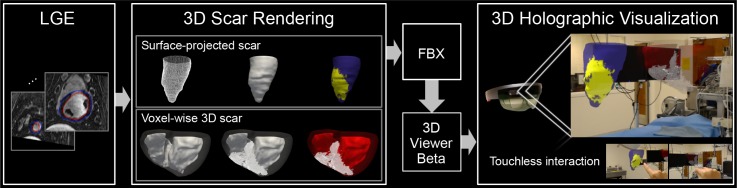
A framework for holographic visualization of the 3D late gadolinium enhancement (LGE) data. Endo- and epicardial contours are delineated in LGE images and surface-projected scar and voxel-wise 3D scar models are generated. 3D models are converted to Filmbox (FBX) file format to be able to load on 3D Viewer Beta (Microsoft, Redmond, WA). 3D scar LGE models are holographically visualized in the 3D mixed-reality space, which provides true 3D perception of the complex scar architecture with immersive experience to explore the 3D LGE images with a touchless interaction while maintaining sterile environment.

We propose a framework for 3D holographic visualization based on the high-resolution 3D LGE images to generate a 3D myocardial scar model and visualize on HoloLens (Microsoft, Redmond, WA). An overview of the proposed framework is presented in Fig 1, and detailed processing pipelines for 3D volumetric and endocardial projection of the scar are presented in the following sections.

### Myocardial geometry reconstruction

The initial step of the framework is reconstructing endo and/or epicardial surface meshes. Previous study showed that reconstructing ventricular geometry using variational implicit function-based method provides the most accurate model [36], therefore we used Poisson surface reconstruction which uses an implicit function framework [37]. Endocardial and epicardial contours were manually delineated from LGE images in all slices. Contours in all slices were merged into a set of point clouds for each surface. Clustering decimation was performed to organize point clouds based on the cell of 3D grid, followed by computing the surface orientation of all vertices based on the 10 nearest neighboring points in the point clouds. Endo/ epicardial triangular surface meshes were then generated using a Poisson surface reconstruction [37, 38].

### Voxel-wise 3D scar rendering

**Fig 2:**
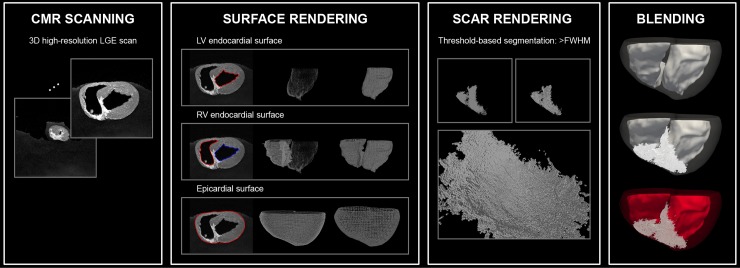
A framework for holographic visualization of the 3D late gadolinium enhancement (LGE) data. Left/ right ventricular (LV/ RV) endo-/ epicardial contours are manually delineated on short-axis view, and LV/RV endo-/ epicardial surfaces are estimated using Poisson surface reconstruction. Scar tissue was defined as voxels in the myocardium with signal intensity greater than a full-width at half-maximum (FWHM). Every voxel was then rendered into a single cube with the actual resolution of the LGE scan. All surface and scar layers were blended with different transparency and color to enhance visibility and perception.

Proposed framework for voxel-wise 3D scar rendered holographic visualization of the high-resolution LGE data is described in Fig 2. Endo and epicardial surface meshes were reconstructed as described in the previous section. Scar tissue was defined as voxels in the myocardium with a signal intensity greater than a full-width at half-maximum (FWHM) [39]. To preserve high-resolution information of LGE voxels, every single voxel of the scar was rendered into a cube which carries the actual resolution of the LGE scan. Any duplicated vertices and faces were removed to reduce the size of the rendered scar mesh. All layers of rendered surface and scar mesh were then blended with different transparency and color to enhance visibility and perception.

### Surface projection of the scar

**Fig 3:**
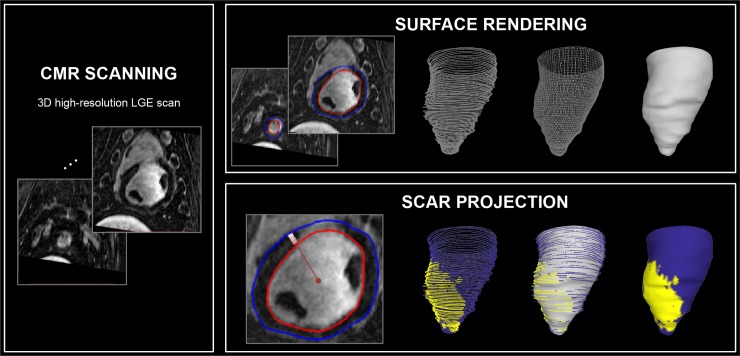
A framework for holographic visualization of endocardial surface with scar projection. LV endo-/ epicardial contours are manually delineated on short-axis view. Endocardial surface is then generated using a Poisson surface reconstruction by first performing clustering decimation followed by the surface orientation estimation based on the 10 neighboring points. To project color-coded scar information on the reconstructed endocardial surface, myocardium was divided into subendo, mid, subepicardial regions, and the subendocardial scar information was projected onto the endocardial surface mesh.

The framework for holographic scar visualization of projected surface model is described in Fig 3. Endocardial surface mesh was first created using a Poisson surface reconstruction as described in the previous section. LGE voxels in the subendocardial region (0–33% from endocardial to epicardial) was projected onto the endocardial points. Endocardial points with projected scar information were then transferred to a texture, which was followed by a texture mapping performed on the parametrized Poisson surface of the endocardial mesh. The proposed framework was implemented in Matlab (MathWorks, Natick, MA) and MeshLab (Visual Computing Lab–ISTI–CNR, Rome, Italy) [38].

### Augmented reality system

Voxel-wise 3D scar rendered model and surface-projected scar model were generated as described in the previous section, which were then exported to Filmbox (FBX) file format on Blender (Blender Foundation, Amsterdam, Netherland) and was imported onto HoloLens using OneDrive (Microsoft, Redmond, WA) and visualized using a 3D Viewer Beta (Microsoft, Redmond, WA).

### System performance

The system performance of the HoloLens while running the generated voxel-wise 3D scar and surface projected scar model were compared with the baseline performance on HoloLens while nothing was running. System on a chip (SoC) power, system power, frame rate, CPU and GPU usage percentage were reported for each case.

### Pilot animal study

A pilot animal study was performed to test feasibility of using holographic 3D LGE visualization in an electrophysiology study. This study was carried out in strict accordance with the recommendations in the Guide for the Care and Use of Laboratory Animals of the National Institutes of Health. The protocol was approved by the Beth Israel Deaconess Medical Center’s Institutional Animal Care and Use Committee (Protocol Number: 100–2014; Boston, MA, USA). All experiments were performed under general anesthesia with isoflurane inhalation (1.5–2.5%) and mechanical ventilation (12–16 breaths/min with tidal volumes between 300-400ml), and animal was euthanized with pentobarbital sodium. Further details regarding animal welfare and ethics are detailed in S1 Supporting Information.

**Fig 4:**
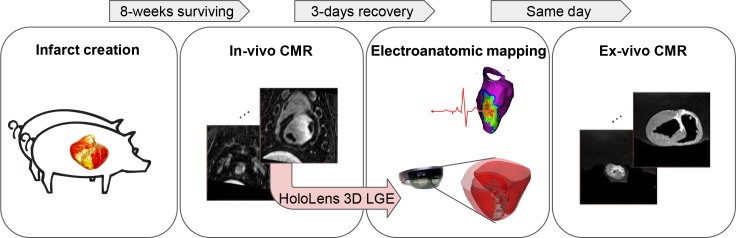
A flowchart of the pilot animal experiment. Myocardial infarction was created by occluding the mid left anterior descending artery. After 8 weeks of recovery and survival periods, animals underwent in-vivo CMR with high-resolution 3D LGE sequence. Based on the in-vivo CMR images, HoloLens 3D LGE model was generated and tested with the electroanatomic substrate mapping. After the electrophysiology study, ex-vivo imaging was performed to create higher-resolution HoloLens 3D LGE model.

A flowchart of the pilot animal experiment is presented in Fig 4. Five swines underwent 180 minute balloon occlusion of the mid left anterior descending artery to create an ischaemia-reperfusion mediated myocardial infarction model [40, 41]. After the infarction, animals were recovered and survived for 8 weeks and underwent in-vivo CMR. High-resolution 3D LGE image was acquired with the low-dimensional-structure self-learning and thresholding reconstruction technique [42–45]. Detailed CMR imaging parameters are reported in the next section. Holographic 3D LGE models were created as proposed using the in-vivo CMR, and after several days of recovery, animals underwent electrophysiology study where VT substrate mapping was performed. After the procedure, ex-vivo CMR imaging was performed and higher-resolution holographic model was generated using the ex-vivo CMR.

In the electrophysiology study, electroanatomic mapping was performed to define the arrhythmogenic VT substrate. Prior to the mapping procedure, both operator and mapping specialist used HoloLens 3D LGE data to review ventricular geometry and myocardial scar architecture, the size and extent of the scar from the voxel-wise 3D scar model and endocardial surface-scar projected model. Upon completion of the study, operator and mapping specialist independently completed the perceived usefulness questionnaire [46] to assess the perceived usefulness of the HoloLens 3D LGE consisting of 6 items on a seven-point Likert scale (1: extremely unlikely– 7: extremely likely). The overall usefulness rating is reported as a mean score of all 6 usefulness items for both the operator and the mapping specialist.

### CMR imaging

In-vivo and ex-vivo CMR imaging were performed to generate holographic 3D LGE models as described in the previous section. CMR imaging was performed using a 1.5 T Philips scanner (Philips Achieva, Best, Netherland) with a 32-element cardiac phased-array receiver coil. For in-vivo imaging, high-resolution 3D LGE images with random under-sampled accelerated acquisition [42–45] were acquired 15–25 minutes after injection of 0.2 mmol/kg gadobenate dimeglumine (MultiHance; Bracco Imaging, Milan, Italy). A respiratory navigator with adaptive acquisition window [47] was used for prospective motion correction. Imaging parameters were as follows: gradient echo imaging sequence; TR/TE = 6.1/2.7 ms; field of view = 270×270×112–280×280×112 mm^3^; flip angle = 25°; isotropic spatial resolution = 1.0×1.0×1.0 mm^3^. Ex-vivo imaging was performed using high-resolution 3D gradient echo sequence with the following imaging parameters: TR/TE = 17/8 ms; field of view = 130×130×100 mm^3^; flip angle = 25°, isotropic spatial resolution = 0.4×0.4×0.5 mm^3^.

## Results

An extensive LGE was observed in the anterior-septal regions in 8 weeks-post myocardial infarction swine. The distribution of the LGE was complex, with the areas of transmural or near transmural scar and subendocardial sparring. The overall LGE volume was 9.2 ± 7.5% of the entire left ventricular myocardial volume, with the endocardial portion of the LGE corresponds to 34.8 ± 8.6% of the total scar volume. The percentage of the LGE area projected on the endocardial surface was 13.8 ± 9.4%. The endocardial LGE surface regions corresponded to the bipolar voltage amplitude < 1.5 mV in the electroanatomic mapping.

For the in-vivo voxel-wise 3D LGE models, the number of vertices were 19,849 ± 13,216 and the number of faces were 81,035 ± 58,794. For the ex-vivo voxel-wise models, the number of vertices were 300,308 ± 382,670 and the number of faces were 962,480 ± 1,311,111. For the surface rendered LGE models, the number of vertices were 7,217 ± 2,381 and the number of faces were 14,263 ± 4,737. The typical processing time of all models for a single animal was about 1–2 hours, most of which was used for performing manual delineation of endo- and epicardial contours. Except for the contour delineation, no process takes longer than several minutes. The overall processing time therefore can be significantly reduced by using automated left and right ventricle segmentation techniques.

The system performance of the HoloLens while running the generated 3D holographic LGE model was compared with the baseline and summarized in Table 1. Surface model that was used for the performance test consists of 7,586 vertices and 2 materials were located. Voxel-wise 3D model that was used for the performance test consists of 290,506 vertices and 3 materials were located. SoC and system power were slightly increased with voxel-wise whole-heart 3D model, but CPU, and GPU usage stayed at the similar level.

**Table 1:** System performance of HoloLens with the 3D LGE models.

	Baseline	Surface-projected Model	Voxel-wise 3D Model
**SoC Power (%)**	56	65	73
**System Power (%)**	60	70	74
**Frame Rate (fps)**	60.05	60.1	60.05
**CPU (%)**	35	34	44
**GPU (%)**	16.81	28.76	25.12

Baseline (when nothing is running on the HoloLens), surface-projected model (when surface projected scar model is running on the HoloLens), and voxel-wise 3D model (when voxel-wise 3D model is running on the HoloLens) were compared for system on a chip (SoC) power, system power, frame rate, CPU, and GPU usage.

**Fig 5:**
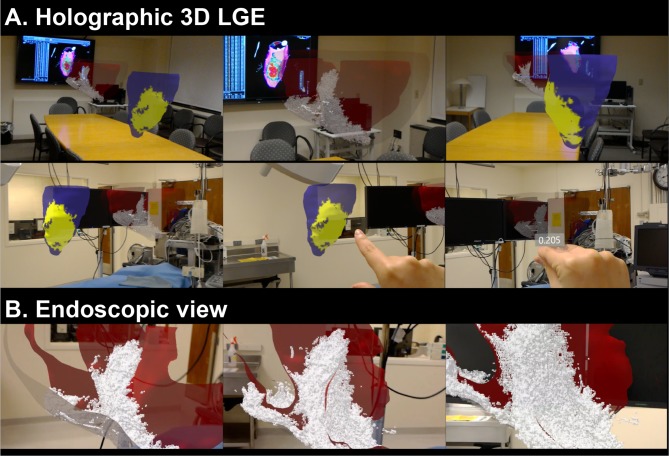
Demonstration of the 3D holographic visualization of the voxel-wise 3D scar rendering and scar projected endocardial surface mesh from 3D LGE data that are generated based on the proposed framework. User can interactively explore 3D LGE myocardial scar by scaling, rotation, moving, and viewing from any perspective in the augmented reality environment (A). In the 3D scar rendered model, as user walks closer to the model, the transparent epicardial surface layer becomes invisible and the inner scar volumes can be explored more in detail (B). Augmented reality representation of the 3D LGE data provides embodied experience to explore the clinical standard LGE images in a more interactive, and interpretable way.

The demonstration of the holographic visualization of the 3D LGE data using the proposed frameworks was recorded on HoloLens and presented in Fig 5 and S1 Video. The 3D holographic visualization of the whole-heart voxel-wise 3D scar rendering and scar projected endocardial surface mesh are demonstrated in Fig 5A. The user could interactively explore 3D LGE myocardial scar by scaling, rotating, moving, and viewing from any perspective in the augmented reality environment that allows for the combination of holographic 3D LGE data interacting with any real-world environments, such as a surgical suite or patient’s body. Holographic 3D LGE data can be viewed in any environment.

Fig 5B represents the endoscopic view of the holographic 3D LGE with the whole-heart voxel-wise 3D LGE model. The user can view the scar from inside of the ventricular chambers. As user steps into the heart, the transparent epicardial surface layer becomes invisible and the inner scar architecture can be explored more in detail.

**Fig 6:**
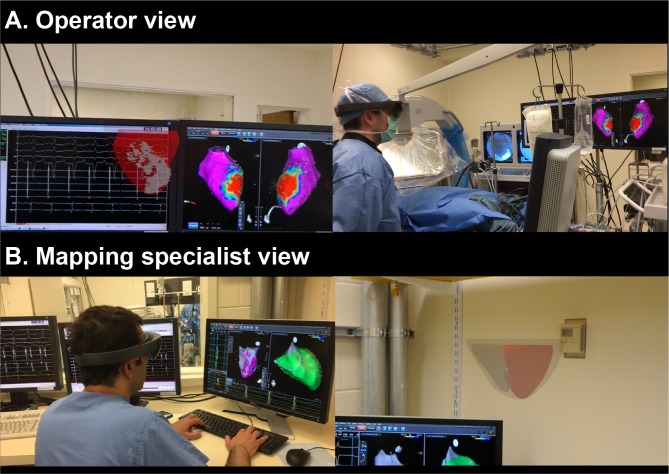
Demonstration of the HoloLens 3D LGE during the electroanatomic mapping and radio-frequency ablation of ventricular tachycardia in the pilot animal study. Holographic voxel-wise 3D LGE was created as proposed using the in-vivo CMR high-resolution 3D LGE images. The holographic LGE models were tested during the electrophysiology procedure. In the operator view, holographic scar can be compared with the electroanatomic data by overlaying holograms on the mapping system (A). The scar information facilitates navigation of electroanatomic mapping procedures (B).

**Fig 7:**
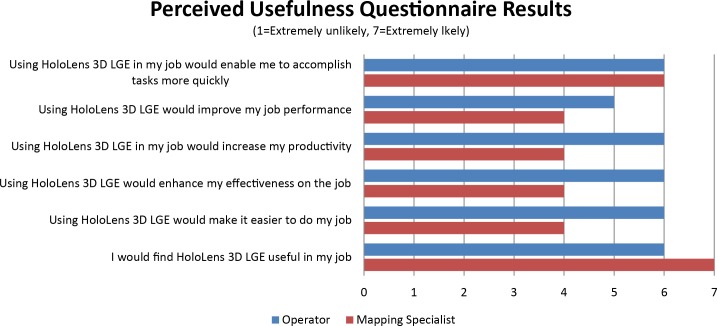
The perceived usefulness questionnaire results from operator and mapping specialist. The usefulness rating is reported as a mean rating of all 6 usefulness items in the questionnaire. Both operator and mapping specialist found HoloLens 3D LGE useful (usefulness rating: operator, 5.8; mapping specialist, 5.5, on a scale from 1 to 7).

Fig 6 shows operator and mapping specialist view during the electrophysiology study. Both operator and mapping specialist found HoloLens 3D LGE useful (overall usefulness rating: operator, 5.8; mapping specialist, 5.5, on a scale from 1: extremely unlikely useful to 7: extremely likely useful; Fig 7) in the VT substrate mapping and RF ablation procedures. The operator could directly review holographic 3D LGE data with a touchless interaction in sterile environment, and without additional hardware installations. The operator could visually compare the detailed 3D scar information with electroanatomic mapping data by overlaying the holographic scar on the electroanatomic map or viewing side by side (Fig 6A). The mapping specialist could review scar information for navigating the electroanatomic mapping (Fig 6B). Examples of operator interacting with HoloLens 3D LGE are presented in S2–S4 Videos.

## Discussion

We present a framework for 3D visualization of the high-resolution 3D LGE of myocardial scar in a holographic manner on the augmented-reality glass HoloLens. High-resolution 3D LGE sequence was used to image the myocardial scar, and 3D rendered LGE model was generated and visualized on HoloLens 3D Viewer Beta. HoloLens 3D LGE was tested in the swine model of myocardial infarction with electrophysiology study of arrhythmogenic substrate mapping. Operator and mapping specialist interacted with HoloLens 3D LGE to review the extent and the structure of 3D myocardial scar and compared with the electroanatomic mapping data. Both operator and mapping specialist found HoloLens 3D LGE useful with a usefulness rating of 5.8 and 5.5, on a scale from 1 to 7, respectively. Holographic visualization of the 3D LGE images provides a true 3D perception of the complex scar architecture with immersive experience to explore the 3D LGE in a more interactive and interpretable way.

Holographic visualization of the LGE images allows to attain touchless interaction with the clinical data in sterile environments, thereby permitting the operator to have increased control and manipulation over pre- or intra-procedural information. Due to a robust gesture tracking using a depth sensor and camera, a user can also operate a HoloLens with a gloved and soiled hand. The availability of such augmented reality technologies to potentially improve procedural efficacy may be useful in the future for complex procedures, and the proposed framework can also be applied in other interventional and surgical procedures.

HoloLens 3D LGE allows detailed scar depiction with a 3D perception. Flat-screen visualizations of the complex three-dimensional myocardial scar structure limit our understanding due to the lack of the depth perception. Understanding the complex 3D scar anatomy using stereoscopic 3D visualization enables operator and mapping specialist to have a clear plan for VT substrate mapping and RF ablation. The endoscopic view further assist operator for a detailed pre-procedural plan for the endocardial catheter mapping.

The current electrophysiology procedures are usually performed as a team of operator and a mapping specialist. Due to the sterility concerns, the operator does not have access to the mapping system inputs, which limits operator’s ability to directly interact with maps during the intervention. HoloLens will provide the operator a higher level of control and visibility and reduce the frustrations of miscommunication between the operator and the mapping specialist by offering touchless interaction. Furthermore, if integrated with the real-time electroanatomic data and the catheter position, it will improve image-based guidance and VT ablation outcome.

Augmented reality allows us to combine holographic 3D LGE data interacting with any real-world environments, and may in the future facilitate data fusion and exploration of the LGE and electroanatomic data to support planning and performing VT ablation. HoloLens also offers unique interactive capabilities between multiple individuals, which may support pre-procedural planning in a group.

Recent study reports significantly reduced procedural and fluoroscopy time when merging LGE into the clinical mapping system to perform VT ablation [19], although another study report no differences in procedural or fluoroscopy time [20]. We did not measure the procedural or fluoroscopy time in this study, however, the impact on having additional information about the myocardial scar may provide similar experience to operators.

We used ex-vivo imaging only for the development phase, and did not test during the intervention or evaluate for the usefulness. Modeling of ex-vivo images can be challenging due to very high spatial resolution (400×400×500 μm^3^ in the current study) and large data size. We showed that three-dimensional holographic visualization of high-resolution ex-vivo images of 400 μm is feasible on HoloLens. Further improvement of the in-vivo CMR imaging techniques will enable higher-resolution imaging and offer a detailed depiction of the myocardial scar in the augmented reality environment during the intervention.

Our animal study was a pilot study towards demonstrating the feasibility of data visualization, and we did not use HoloLens 3D LGE for guiding ablation in real-time. We did not use ablation success as an endpoint in our study. The questionnaire was performed by one operator and one mapping specialist; however, neither of the investigators were involved in developing the technology. The weight of the HoloLens is 579 g which may hamper interventional applications, and further design modification should be done to reduce weight and thereby improve comfort level during the intervention.

## Supporting information

S1 Video Demonstration of the holographic visualization of the 3D LGE data using the proposed frameworks recorded on HoloLens. (MP4)

S2 Video Example of operator interacting with HoloLens 3D LGE. (MP4)

S3 Video Example of operator interacting with HoloLens 3D LGE. (MP4)

S4 Video Example of operator interacting with HoloLens 3D LGE. (MP4)

S1 Supporting Information Animal welfare and ethics. (PDF)

S1 Dataset HoloLens models used in this study. (ZIP)

S2 Dataset HoloLens models used in this study. (ZIP)
