# Supplementary material for: Three-dimensional holographic visualization of high-resolution myocardial scar on HoloLens
Source: PLoS One. 2018 Oct 8;13(10):e0205188. doi: 10.1371/journal.pone.0205188 (PMC6175509; doi:10.1371/journal.pone.0205188)
Supplement: S1 Supporting Information — (PDF) [file pone.0205188.s005.pdf]

S1 Supporting Information. Animal welfare and ethics.

Ethics committee protocol

The Beth Israel Deaconess Medical Center's Institutional Animal Care and Use Committee, Boston, MA. USA, reviewed and approved the protocol of this study accordance with the recommendations in the Guide for the Care and Use of Laboratory Animals of the National Institutes of Health. The institution holds an OLAW Assurance (D16-00093(A3153-=1)), an USDA registration (14-R-0138) and has Full Accreditation with Association for Assessment and Accreditation of Laboratory Animal Care (unit # 000179- March 30, 2017).

Animal welfare

Swine are housed in pens allowing for 2.5-5 m^2 of floor space, based on the animals size/weight and the standards set forth in the Guide For the Care and Use of Laboratory Animals. All animal room temperatures are maintained between 20-25°C degrees with humidity between 30-70%. Swine are fed LabDiet Porcine Lab Grower Diet # 5084. Potable tap water is supplied using automatic water devices. Environmental enrichment is provided daily in the form of human contact, exercise outside of the pen during sanitation, as well as from enrichment devices purchased specifically for the species.

Post-operative care and monitoring

Animals are monitored immediately post-operatively until sternal then additionally for several hours until the animal is standing and walking. Post-operative care continues at least twice daily for a minimum of 5 days, or longer if needed. All USDA covered species are monitored,

regardless of the surgical status, at least once daily by the Veterinary Technical staff, with additional visits from the research staff. Analgesia is provided using Buprenorphine (0.01-0.05 mg/kg) administered during surgery, as well as Fentanyl transdermal patches (2-4 ug/kg/hr) with duration of 72 hours. A full-time attending Veterinarian oversees the surgical program.

Humane endpoints

Any animal that appears to experience unrelieved discomfort will be euthanized prior to expected study endpoint. This includes any of the following symptoms without relief during the survival period will be euthanized: decreased appetite, labored breathing, respiratory distress, pain/discomfort. The ARF staff should contact the study team if animal well-being is a concern, as no party wishes an animal in this study to be in untreatable discomfort or distress. Euthanaisia will be performed with Fatal Plus. Animals are euthanized upon study completion during terminal mapping procedure performed under general anesthesia and analgesia.
